# Anticancer Activity of Biogenic Selenium Nanoparticles: Apoptotic and Immunogenic Cell Death Markers in Colon Cancer Cells

**DOI:** 10.3390/cancers13215335

**Published:** 2021-10-24

**Authors:** Katerina Spyridopoulou, Georgios Aindelis, Aglaia Pappa, Katerina Chlichlia

**Affiliations:** Department of Molecular Biology and Genetics, Democritus University of Thrace, University Campus Dragana, 68100 Alexandroupolis, Greece; aspyrid@mbg.duth.gr (K.S.); gaindeli@mbg.duth.gr (G.A.); apappa@mbg.duth.gr (A.P.)

**Keywords:** biogenic nanoparticles, selenium nanoparticles, SeNps, *Lactobacillus casei*, colon cancer, apoptosis, immunogenic cell death

## Abstract

**Simple Summary:**

Colorectal cancer remains a major health problem worldwide, with high prevalence and mortality rates. Innovative approaches and advanced therapeutic anticancer strategies need to be developed. In this frame, the aim of the present study was to assess the potential of biogenic selenium nanoparticles, produced by a probiotic microorganism, to induce apoptotic and immunogenic cell death in colon cancer cell lines. The findings of the present study confirmed that treatment of colon cancer cell lines with selenium nanoparticles induced pro-apoptotic and immunogenic cell death markers (indicating immunogenic cell death) and highlighted their potential to provide an efficient strategy for destroying tumour cells not only directly by apoptotic cell death, but also indirectly through enhanced immunogenicity.

**Abstract:**

Colorectal cancer is a health problem with high mortality rates and prevalence. Thus, innovative treatment approaches need to be developed. Biogenic nanoparticles are nanomaterials that can be synthesised in biological systems and, compared to chemically synthesised nanoparticles, have better bioavailability while being more cost-effective, eco-friendlier, and less toxic. In our previous studies, the probiotic strain *Lactobacillus casei* ATCC 393 was used to synthesise selenium nanoparticles (SeNps), which were shown to inhibit colon cancer cell growth in vitro and in vivo. Herein, we have further investigated SeNps’ pro-apoptotic activity and their ability to induce immunogenic cell death (ICD) in colon cancer cells. The SeNps’ effect on Caco-2 cells growth was examined along with their potential to induce caspase activation. Moreover, the expression of typical pro-apoptotic and ICD markers were examined in SeNps-treated HT29 and CT26 cells by flow cytometry, Western blot, ELISA and fluorescence microscopy. Elevated caspase-3 activation and surface phosphatyldoserine, that subsided upon co-incubation with a pan-caspase inhibitor, were detected in SeNps-treated cells. Furthermore, nanoparticles induced modulation of the expression of various apoptosis-related proteins. We also report the detection of biomarkers involved in ICD, namely the translocation of calreticulin and ERp57, the release of HMGB1 and ATP, and the secretion of pro-inflammatory cytokines from SeNps-treated cells. Moreover, RAW246.7 macrophages exhibited a higher rate of phagocytosis against treated CT26 when compared to control cells. Taken together, our findings indicate that treatment with SeNps might be an efficient strategy to destroy tumour cells by inducing apoptotic cell death and triggering immune responses.

## 1. Introduction

Selenium is a nonmetal, essential trace element for humans with various health benefiting effects, such as anti-oxidant, anti-inflammatory and anticancer activities [1,2,3,4]. Active selenium compounds come in many different forms, such as selenoproteins, selenium salts and nanoparticles [5,6,7], with their chemical forms being a crucial element of their beneficial properties [8]. Noteworthy, selenium compounds have been reported to possess both anti-oxidant as well as pro-oxidant properties, depending on the chemical form and the concentration/dose of the selenium compound used [9]. Selenium nanoparticles in particular have emerged as a candidate for the implementation of selenium into clinical practice due to their unique physicochemical characteristics, such as their high biocompatibility, stability and bioavailability, as well as their low toxicity [7,10,11,12]. Up until now, chemical procedures have been the cornerstone of selenium nanoparticles synthesis. However, in recent years, the biosynthesis of nanoparticles utilising microorganisms has been attracting attention as a cheaper, simpler, and environmentally friendly alternative. Biogenic nanoparticles’ surfaces are often associated with biomolecules derived from the microorganism that has been used for their synthesis. These biomolecules comprise the surface layer that prevents aggregation, enhances stability and leads to a more bioavailable and less toxic profile. Moreover, the use of microorganisms excludes the use of harmful chemicals during the synthesis process [13,14]. It is noteworthy that the enzymatic nature of the biosynthetic routes results in a variety of formulations among different strains, and a well-controlled specificity of the nanoparticles’ characteristics, and therefore bioactivities, that are beyond those that can be achieved by either chemical or physical synthesis methods [15,16].

Out of the many biological and health beneficial effects of selenium nanoparticles, their antiproliferative and anticancer properties may hold promise for the development of novel approaches in cancer prevention and treatment. Several studies have highlighted the ability of selenium nanoparticles to selectively induce apoptosis in various types of cancer cells [12], while protecting normal healthy cells from damage [17,18]. The mechanisms through which selenium nanoparticles destroy cancer cells are not yet fully understood, however, the most common pathways involve internalization of the nanoparticles, regulation of the production of reactive oxygen species, induction of autophagy and activation of the intrinsic apoptotic machinery [17,19]. Having noted that, while the antiproliferative and pro-apoptotic activities of selenium nanoparticles are extremely important for achieving a direct effect in destroying cancer cells, additional mechanisms appear to be involved in their anti-tumour activity. 

Activation of the immune system and its constituents is of utmost significance during cancer treatment [20,21]. Immunogenic cell death (ICD) is a type of programmed cell death, accompanied by a pattern of distinct, adjuvant signals driven by organellar and cellular stress [22,23]. These signals are molecules called damage-associated molecular patterns (DAMPs), and their release and subsequent binding to pattern recognition receptors of dendritic cells culminates in the activation of innate and adaptive immunity [24,25]. In fact, recent evidence has revealed that the ICD of tumour cells, due to treatment with ICD-inducing agents, can trigger enhanced anticancer immune responses that boost the effects of traditional chemotherapy and radiotherapy [20,21,26,27]. Irinotecan, oxaliplatin, doxorubicin, 5-fluorouracil, mitoxantrone and anthracyclines are traditional chemotherapeutic agents that have been proven to induce ICD in colon cancer cells in both preclinical in vitro and in vivo models [28,29]. However, only a small number of agents that elicit ICD have been identified and tested in the clinical setting [21,30]. These few agents are of particular importance, as they can be an initiating step in the development of combinatorial anticancer treatment schemes with various forms of immunotherapy and thus, considerable attention has been devoted to their research, evident by the increasing number of animal and human studies [31,32,33,34,35]. Besides the direct cytotoxic effects exerted by selenium nanoparticles against tumour cells, these nanoparticles have also been shown to affect immune regulation, a property which could be extremely useful if employed in the development of novel anticancer strategies [19,36]. It is noteworthy that biogenic selenium nanoparticles have been reported to exert an immunostimulatory effect in a murine BALB/c 4T1 breast cancer model, resulting in an inhibition of tumour growth and a longer survival rate [37]. Moreover, selenium nanoparticles have been shown to induce tumour growth inhibition by exerting immunomodulatory effects that lead to the enhancement of anti-tumour immune responses, such as the regulation of tumour-associated macrophages and the activation of specific T-cells [19,38,39,40]. 

We had previously reported that the probiotic strain *Lactobacillus casei* ATCC 393 is able to synthesise selenium nanoparticles (SeNps), and described the synthesis and extraction methods as well as the characterisation of the nanoparticles. These nanoparticles were found to be biocompatible and to exert cancer-specific growth-inhibitory effects, evident by the suppression of colon cancer cell growth in vitro and in vivo upon oral administration in a CT26 mouse colorectal cancer model. Moreover, it was shown that SeNps trigger ROS generation in HT29 cancer cells, contributing possibly to the eventual induction of apoptosis [41]. In this study, we aimed to further investigate the pro-apoptotic and the potential immunostimulatory activity of the SeNps against colon cancer cells. For this purpose, we analysed the expression of typical pro-apoptotic markers and examined the induction of ICD by identifying characteristic markers of this cell death modality in colon cancer cells of human (HT29) and mouse (CT26) origin, following SeNps treatment. Our findings further confirm that SeNps destroy colon cancer cells by inducing the activation of the apoptotic machinery and that, in addition, SeNps elicit the generation and emission of distinct DAMPs that have been shown to act as ancillary signals which can enhance immunogenicity and may give rise to tumour-specific adaptive immune responses.

## 2. Materials and Methods

### 2.1. Materials

Dulbecco’s Modified Eagle’s Medium (DMEM) and Fungizone (Amphotericin B) were purchased from Gibco (Waltham, MA, USA). Fetal bovine serum (FBS), trypsin, penicillin/streptomycin, and phosphate-buffered saline (PBS) were purchased from Biosera (Boussens, France). MRS Broth, LAB094 was purchased from LabM (Bury, UK). The Annexin V/PI kit was purchased from BD Biosciences (Franklin Lakes, NJ, USA). Acetic acid, trichloroacetic acid (TCA), Trizma base, sulforhodamine B (SRB), NaHSeO_3_ and all other chemicals mentioned were purchased from Sigma-Aldrich (St. Louis, MO, USA).

### 2.2. Cell lines and Bacterial Cultures

Colon carcinoma cells of mouse (CT26) or human (HT29 and Caco-2) origin and a murine macrophage cell line RAW264.7 were maintained in a humidified incubator with 5% CO_2_ at 37 °C. Cells were regularly sub-cultured under sterile conditions and grown in DMEM, supplemented with 10% FBS, 100 U/mL penicillin, 100 μg/mL streptomycin and 2 mM glutamine. Cell lines were obtained from ATCC.

The *Lactobacillus casei* ATCC 393 bacterial strain was used for the synthesis of SeNps as we have previously described [41]. Briefly, bacteria were inoculated at a concentration of 10^7^ CFU/mL into MRS broth, supplemented with 20 μg/mL NaHSeO_3_ as a selenium source and incubated at 37 °C without agitation for 96 h. Bacterial cells were collected after centrifugation at 1700× *g* for 15 min at 4 °C and the synthesised SeNps were extracted and purified.

### 2.3. Selenium Nanoparticles

The nanoparticles used in this study were extracted from *L. casei* ATCC 393 bacteria, grown in the presence of NaHSeO_3_ and characterised according to the methods we have previously described [41]. As concluded in our previous study, nanoparticles synthesised by *L. casei* ATCC 393 under these experimental conditions, are red, amorphous and spherical selenium nanoparticles with a mean diameter of 360 nm [41].

### 2.4. TEM Analysis

LC and LCSe bacteria were observed under a high resolution JEM 2100 transmission electron microscope (JEOL), at an operating voltage of 80 kV. The samples were fixed in a 2.5% glutaraldehyde solution at 4 °C overnight, and then rinsed with sodium cacodylate buffer SCB (0.1 M, pH 7.2) for 15 min. The rinsing step was repeated an additional two times. Samples were then fixed with 1% osmium tetroxide for 2 h at RT and rinsed with the 0.1 M SCB buffer three times for 15 min each time. The fixed samples were dehydrated with ethanol solutions with gradient concentrations (30%, 50%, 70%, 80%, 90%, 95%, 100%) for 15 min for each concentration, and treated with 100% dry ethanol once for 20 min, followed by immersion into propylene oxide 2 times for 15 min each time. The samples were then treated with a mixed solution of resin-embedding media and propylene oxide (*v:v* = 1:3) for 1 h, then with a mixed solution of resin-embedding media and propylene oxide (*v:v* = 1:1) for 1 h, a mixed solution of resin-embedding media and propylene oxide (*v:v* = 3:1) for 1 h, and, finally, with 100% resin-embedding media overnight. Afterwards the samples were heated at 60 °C for 48 h. Sections were cut on a Ultramicrotome LEICA EM UC7 at a thickness of 70 nm and then picked up on copper, 300 mesh grids and stained with lead citrate and uranyl acetate.

### 2.5. Micro Fourier Transform Infrared Spectroscopy

The surface chemistry of isolated SeNps was analysed by micro-Fourier transform infrared spectroscopy (μ-FTIR). The analysis was performed with an i-Series Perkin-Elmer FTIR microscope, equipped with a nitrogen cooled MCT detector and coupled with a Perkin-Elmer spectroscope, model Spectrum 1000. A small quantity of the SeNps was placed on a freshly prepared KBr pellet and flattened using a roller blade knife tool. The spectra were collected in the mid IR area (4000–400 cm^−1^), in transmittance mode, with 32 scans and a resolution of 4 cm^−1^.

### 2.6. Cell Growth Assay

Cell viability was determined by the SRB [42] assay. Caco-2 cells were seeded in 96-well plates at a cell density of 4000 cells per well. Cells were left to adhere overnight and treated with the indicated concentrations of SeNps for 24 or 48 h. Next, cells were fixed, dried and stained as previously described [41]. For the quantification of the optical density, a microplate reader (Enspire, Perkin Elmer, Waltham, MA, USA) was used. Background levels due to the presence of SeNps were excluded. The inhibition of cell growth percentage was calculated by the following formula:

% growth inhibition= 100 − [ (mean OD sample)/(mean OD control) × 100]

### 2.7. Western Blot

Cancer cells were seeded in 100 mm plates at a density of 1.5 × 10^6^ for CT26 cells and 2 × 10^6^ for HT29 cells per plate, were left overnight to adhere and then treated with 15 μg/mL of SeNps. Non-treated cells were used as controls. For the isolation of total protein extracts from cells, RIPA buffer was added to the cell pellet and samples were incubated on ice for 30 min, with occasional pipetting and a subsequent centrifugation at 14,000× *g*, at 4 °C for 20 min. For fractionation of cytoplasmic and nuclear proteins, lysis buffer 1 (10 mM HEPES pH 7.9, 10 mM KCl, 0.1 mM EDTA, 1.5 mM MgCl2, 0.2% *v*/*v* NP40) was added to the cell pellet. Samples were left on ice for 10 min with occasional gentle pipetting and then centrifuged at 1000× *g*, at 4 °C for 10 min. Cytoplasmic proteins which had dissolved in the supernatant were collected and the pellet was washed twice with lysis buffer 1, before the addition of lysis buffer 2 (20 mM HEPES pH 7.9, 420 mM NaCl, 0.1 mM EDTA, 1.5 mM MgCl_2_, 25% *v*/*v* glycerol) and incubation on ice for 10 min with frequent pipetting. Samples were then centrifuged at 14,000× *g*, at 4 °C for 10 min and the supernatant was collected. Concentration of the proteins was determined by the bicinchoninic acid (BCA) protein assay kit (Invitrogen, Waltham, MA, USA). Equal amount of the protein extracts were loaded onto SDS polyacrylamide gels. Separated proteins were transferred onto 0.45 μm or 0.22 μm PVDF membranes according to molecular weight. Non-specific sites were blocked with 5% *w*/*v* non-fat dry milk and then the membranes were incubated overnight, with primary antibodies in blocking buffer, at 4 °C. Next, the membranes were incubated with a horseradish peroxidase (HRP)-conjugated secondary antibody (1:2000) in blocking buffer for 1 h at room temperature. Bands were detected by autoradiography with an ECL HRP chemiluminescent substrate after exposure to Kodak films. Beta-tubulin and histone H3 were used as loading controls.

### 2.8. Analysis of Apoptosis with Annexin V/PI Staining

A commercially available kit, utilising the Annexin V/PI double staining assay, was used for the detection of apoptotic cell death. Cancer cells were seeded in 6-well plates at a density of 0.25 × 10^6^ for CT26 or 0.35 × 10^6^ for HT29 cells per plate. Seeded cells were left overnight to adhere and were then treated with 15 μg/mL of SeNps for the indicated time periods. For the co-treatment with the pan-caspase inhibitor zVAD-FMK (zVAD) (tlrl-vad, Invivogen), cells were exposed to 20 μΜ of zVAD for 1 h before the addition of SeNps. Untreated cells were used as controls. Following treatment, cells were harvested using 7.5 mM EDTA in PBS and suspended in Annexin binding buffer. Cells were stained with Annexin V-FITC and PI for 15 min at room temperature in the dark and analysed with an Attune NxT flow cytometer. Data analysis was performed with FlowJo V10 software (FlowJo LCC, Ashland, OR, USA).

### 2.9. Detection of Caspase 3 Clavage by Flow Cytometry

Cancer cells were seeded in 100 mm plates at a density of 1.5 × 10^6^ for CT26 and 2 × 10^6^ for HT29 cells per plate, left overnight to adhere and were then treated with 20 μM zVAD as described above and/or 15 μg/mL SeNps for 21 h. Control cells were cultured in DMEM. Following treatment, cells were washed once and collected with PBS containing 7.5 mM EDTA. Cytofix/Cytoperm Fixation/Permeabilization kit (554714) from BD Biosciences was used to permeabilise cells, according to manufacturer’s instructions. Cells were then labelled with an anti-cleaved caspase 3 (9661, Cell Signaling, Danvers, MA, USA) antibody (1:1000) for 45 min on ice, washed twice with Perm/Wash buffer and subsequently stained with an anti-rabbit Alexa Fluor 488-conjugated (A11008, ThermoFischer, Waltham, MA, USA) secondary antibody (1:1000) for 40 min on ice. Samples were again washed twice with Perm/Wash buffer, suspended in PBS, and analysed with an Attune NxT flow cytometer. Data analysis was performed with FlowJo V10 software.

### 2.10. M30 Apoptosense Assay

Apoptosis was also measured with the M30 Apoptosense^®^ ELISA kit (Peviva AB, Stockholm, Sweden) in Caco-2 cells, as previously described [43]. The M30 Apoptosense assay quantifies the soluble apoptosis-related and caspase-cleaved K18 (ccK18) fragments containing the K18Asp396 neo-epitope. This epitope becomes accessible only after the proteolytic processing of K18 by caspases 3/7/9. The method is a solid-phase sandwich enzyme immunoassay, where the samples react with an anti-K18 solid phase capture antibody, the HRP (horseradish peroxidase) conjugated M30 antibody, which is directed against the K18Asp396 neo-epitope. The measurement of absorbance at 450 nm with a spectrophotometer is directly proportional to the concentration of the analyte.

### 2.11. Calreticulin and ERp57 Exposure Analysis by Flow Cytometry

Cancer cells were seeded in 6-well plates at a density of 0.25 × 10^6^ for CT26 and 0.35 × 10^6^ for HT29 cells per plate, left overnight to adhere and then treated with 15 μg/mL SeNps for the indicated time periods. Control cells were cultured in DMEM. Treated cells were washed once with PBS, detached from plates with PBS containing 7.5 mM EDTA, and centrifuged at 500× *g*, at 4 °C for 5 min. Cells were then stained with anti-calreticulin (ab2907, 1:500) or anti-ERp57 (ab10267, 1:1000) primary antibodies in FACS buffer (PBS, 2.5% FBS, 2.5 mM EDTA) for 45 min on ice, washed twice with FACS buffer and then stained with anti-rabbit Alexa Fluor 488-conjugated secondary antibody (1:1000) for 40 min on ice. Stained cells were again washed twice with FACS buffer, suspended in PBS, and then 7-aminoactinomycin D (7-AAD, 559925, BD) was added 5 min prior to the analysis with an Attune NxT flow cytometer. Dead cells which were positive for 7-AAD were excluded from the analysis (see Figure S1 for gating strategy). Data analysis was performed with FlowJo V10 software (Tree Star, Inc., Ashland, OR, USA).

### 2.12. Flow Cytometry Analysis of Intracellular ATP

Cells were seeded in 6-well plates as described above and treated with SeNps (15 μg/mL) for the indicated time points. Next, cells were harvested with 7.5 mM EDTA in PBS, washed, and labelled with 4 μΜ quinacrine dihydrochloride (ab120749, Abcam, Cambridge, UK) for 30 min. Cells were washed, incubated with 7-AAD for 5 min for the exclusion of 7-AAD positive, and therefore dead, cells from the analysis, and then were analysed with an Attune NxT flow cytometer (see Figure S1 for gating strategy). Data analysis was performed with FlowJo V10 software.

### 2.13. Detection of Annexin V / PI Staining, HMGB1 Translocation, Caspase 3 Cleavage and ATP Depletion by Fluorescence Microscopy

Cancer cells were seeded onto glass coverslips, left overnight to adhere and then treated with 15 μg/mL SeNps for 24 h. Cells cultured in DMEM were used as controls. Cells were washed once with PBS and then fixed with 4% PFA for 10 min. Coverslips were washed three times with PBS for 5 min each, and then the fixed cells were permeabilised with 0.2% Triton-X 100 in PBS for 10 min. Following permeabilisation, coverslips were blocked with 2% FBS in permeabilisation buffer and then stained with primary antibodies against cleaved caspase 3 (1:200) or HMGB1 (1:500) (ab18256, Abcam) for 1 h. After three subsequent washes with PBS, cells were incubated with anti-rabbit Alexa Fluor 488-conjugated secondary antibody (1:1000) for 30 min. Following another three washing steps, nuclei were stained with Hoechst (ThermoFischer) for 15 min at room temperature, the excess dye was washed away and, finally, the coverslips were mounted onto glass slides. For the visualization of ATP-containing vesicles and Annexin V/PI staining, live cell imaging was performed. Cells were seeded onto either an 8-well μ-slide (IBIDI, Gräfelfing, Germany) or onto coverslips and grown for 24 h before being treated with 15 μg/mL SeNps for 21 (ATP) or 24 (Annexin V/PI) hours. Before being visualized under the fluorescence microscope, cells were stained with 4 μM quinacrine for 20 min and thoroughly washed with PBS [44,45] or incubated with Annexin V-FITC and PI as described above. Cells were imaged on a Zeiss Axio Scope A1 microscope and image acquisition was performed using the ZEN Blue imaging software by Carl Zeiss Microscopy. For the quantification of nuclear HMGB1 and quinacrine (ATP-containing vesicles) [46] levels in cells, images were analysed with ImageJ (1.50i, NIH) according to the method previously described [47,48]. Briefly, an outline was drawn around individual cells for quinacrine, nuclei for HMGB1, and around adjacent cell-free regions for the determination of the background signal. The area, integrated density and mean grey values were measured. The corrected total cell cellular fluorescence (CTCF) was calculated with the following equation:

CTCF = Integrated Density − (Area of selected cell × Mean fluorescence of background readings)

Calculated CTCF values were normalized to CTCF values of the controls, the untreated cells, and reported as the percentage of relative fluorescence intensity (FI).

### 2.14. Detection of Apoptosis Related Proteins in CT26 Cells with a Membrane-Based Sandwich Immunoassay

A commercially available kit (ARY031, R&D Systems, MN, USA) was used for the detection of various apoptosis-related proteins. CT26 cells were seeded at a density of 1.5 × 10^6^ cells per plate, left overnight to adhere and then treated with 15 μg/mL of SeNps for 24 h. Untreated cells were used as controls. Following treatment, cells were washed once with PBS, harvested using EDTA, and subsequently, the manufacturer’s supplied lysis buffer, supplemented with protease inhibitors, was added to the cell pellet. Samples were incubated on ice for 30 min, rocking gently with occasional pipetting, and then centrifuged for 5 min at 14,000× *g* at 4 °C and the supernatant-containing, isolated proteins were collected and stored at −80 °C. Protein concentration of the samples was calculated by the bicinchoninic acid (BCA) protein assay kit, and 400 μg of proteins from each sample were added to the kit membrane. The procedure was performed according to manufacturer’s instructions and the signal was captured with a Chemidoc XRS system. For original images of the analysed dot plots see Figure S2. Data analysis was performed with ImageJ.

### 2.15. Quantification of Cytokines in SeNps-Treated Cells Supernatants

The levels of IL-6, IL-8 and TNF-α were measured in SeNps-treated HT29 and/or CT26 culture supernatants. Cells were seeded in 96-well plates (10,000 cells per well for HT29 and 5000 cells per well for CT26), left to adhere for 24 h, and treated with 15 μg/mL SeNps for 3 h. Quantification of cytokines in the culture supernatants was performed by ELISA. Results are presented as the mean value ± S.D (standard deviation) of at least three independent experiments. Culture supernatants were collected, and their cytokine levels were quantified with the sandwich enzyme-linked immunosorbent assay.The sandwich ELISA is a qualitative method that detects antigens between two layers of antibodies (i.e., capture and detection antibody). For each cytokine, a different ELISA kit (eBioscience, San Diego, CA, USA) was used according to the manufacturer’s protocols (mouse TNF-α: 88-7324-22, human IL-6: 88-7066, mouse IL-6: 88-7064-22, mouse IL-8: 88-8086-22. The O.D. (optical density) of the reaction products was measured in an ELISA plate reader (EnSpire Multimode Plate Reader). Data were analysed with Sigma Plot Software v.11 (Systat Software GmbH, Erkrath, Germany). 

### 2.16. Assessment of Phagocytic Activity in Macrophages and SeNps-Treated Tumour Cells Co-Culture

The LPS-stimulated RAW264.7 murine macrophage model was used in order to assess the phagocytic activity of murine RAW264.7 macrophages against SeNps-treated CT26 cells. RAW264.7 cells were seeded in glass 60 mm dishes (1 × 10^6^ cells/dish) and left to adhere overnight. Next, cells were stimulated with 1 μg/mL LPS (L4391, Simga-Aldrich) for 24 h. CT26 cells were seeded in 48-well plates at a density of 3 × 10^4^ cells/well, left to adhere overnight, and then were stained with carboxyfluorescein succinimidyl ester (CFSE, CellTrace CFSE Cell Proliferation kit, Invitrogen) according to the manufacturers’ instructions, before being treated with 15 μg/mL SeNps for 48 h. RAW264.7 macrophages (LPS-stimulated) and CT26 cells were harvested with Trypsin (Biosera) and counted. RAW264.7 cells were stained with CellBrite^®^ Red (Biotium, Hayward, CA, USA) according to the manufacturer’s instructions. Red-labelled RAW264.7 macrophages and green-labelled SeNps-treated CT26 cells were co-cultured at a cell ratio 2:1 in DMEM, in a humidified incubator with 5% CO_2_ at 37 °C for 3 h. Cells were collected, washed in PBS and analysed with an Attune NxT flow cytometer. Data collected were analysed with FlowJo V10 software (Tree Star, Inc.). The results presented are representative of three experiments.

### 2.17. Data Analysis and Statistics 

All data are representative of at least three independent experiments, unless stated otherwise. Microscopy images and Western blots were analysed with ImageJand flow cytometry data with FlowJo v.10. All resulting data were analysed with Sigma Plot v.11.0. Statistical comparisons between groups were performed using the Student’s *t*-test. Differences between groups were considered statistically significant when *p* < 0.05 (* *p* < 0.05, ** *p* < 0.01, *** *p* < 0.001).

## 3. Results

### 3.1. Characteristics of SeNps

**Figure 1:**
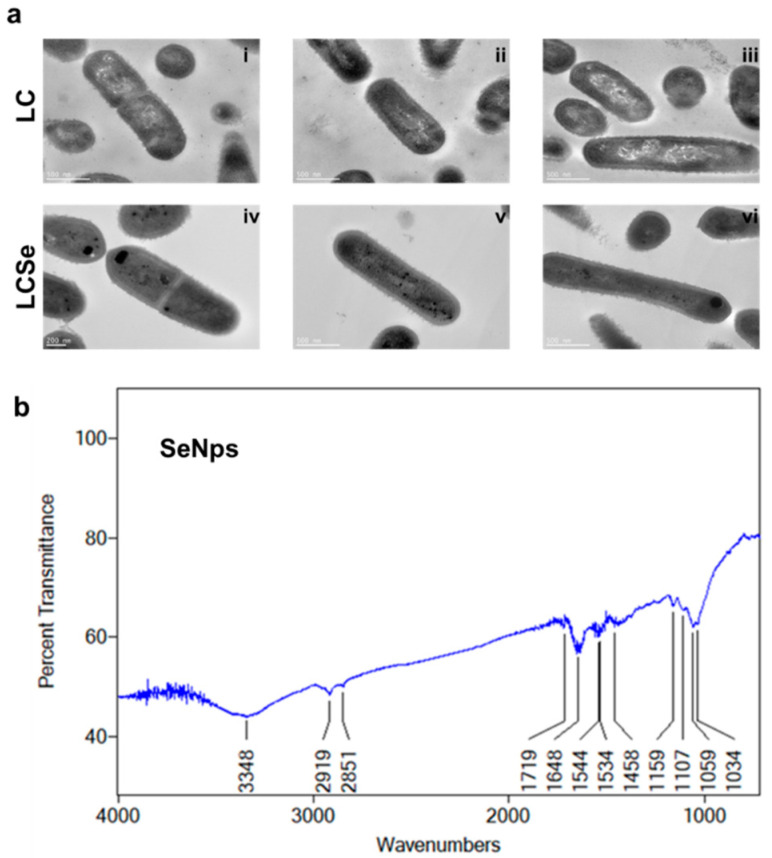
Characteristics of SeNps. (**a**) TEM images of *L. casei* ATCC 393 bacteria grown in their standard medium (LC) (i–iii) or in the presence of 20 μg/mL NaHSeO_3_ (LCSe) (iv–vi). Note the dark spots present only in LCSe, indicating the intracellular formation of SeNps (magnification: 10,000×). (**b**) μ-FTIR spectrum of biogenic SeNps synthesised by *L. casei ATCC* 393.

SeNps were found to be biosynthesised intracellularly by *L. casei* ATCC 393 bacteria as was made evident by TEM analysis. Distinct intracellular dark spots were observed only in the LCSe bacteria grown in the presence of NaHSeO_3_ (Figure 1a(iv–vi)), and not in the control LC bacteria that were cultured in their standard medium (Figure 1a(i–iii)).

The μ-FTIR spectrum of isolated SeNps shows the presence of certain biomolecules originating from the bacteria, on the nanoparticles’ surfaces (Figure 1b). The band at 3348 cm^−1^ corresponds to the stretching vibration peak of O-H and N-H [49,50]. The bands at 2919 and 2851 cm^−1^ indicate the stretching vibrations in C-H [49,51]. The band at 1719 cm^−1^ is associated with the stretching vibrations of C = O [50]. The band at 1648 cm^−1^ corresponds to the amide I region, while the bands at 1544 and 1534 cm^−1^ correspond to the amide II region [51,52]. The band at 1458 cm^−1^ is assigned to COO- residues [50], while the bands at 1159, 1107, 1059 and 1034 cm^−1^ fall within the typical polysaccharide vibration region 1200–1000 cm^−1^ [51,52]. These data indicate that SeNps might be capped with bacteria-derived biomolecules, such as proteins, lipids and polysaccharides. Moreover, the μ-FTIR analysis of the LC and LCSe microbial dried biomass (Figure S4) revealed quite similar spectra that include the typical bands for bacterial FTIR spectra [53].

### 3.2. Induction of Apoptosis in Colon Cancer Cells Treated with SeNps

#### 3.2.1. SeNps Induce Apoptosis in Human Colon Cancer Cells

**Figure 2:**
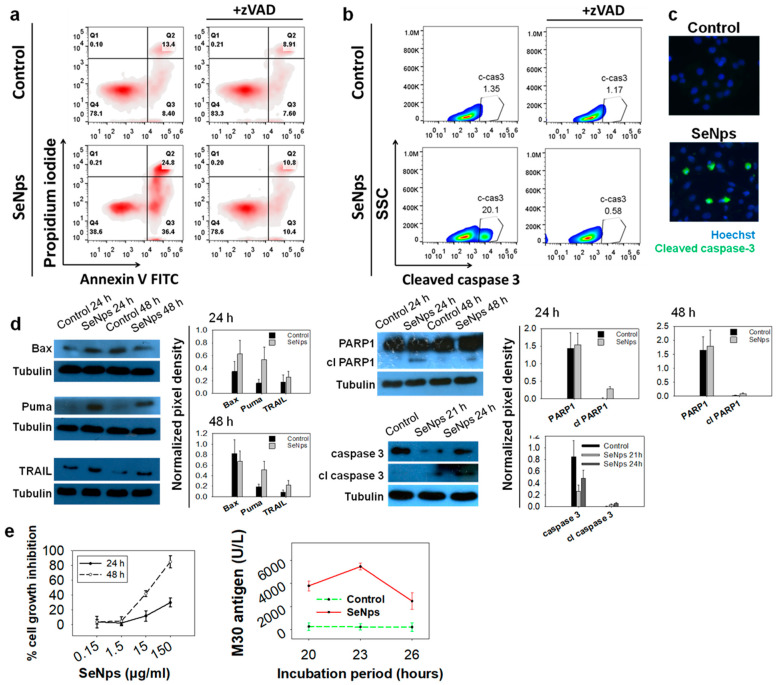
SeNps induce apoptosis in human colon cancer cells. (**a**) SeNps induce apoptotic cell death in HT29 cells which was reversed by the pan-caspase inhibitor zVAD-FMK. Flow cytometry density plots of control and treated cells. The percentages of viable (Annexin V-FITC and PI-negative), early apoptotic (Annexin V-positive and PI-negative), and late apoptotic/necrotic (Annexin V/PI-double-positive) cells are indicated on the plots. Cells were treated with 15 μg/mL SeNps for 24 h and stained with Annexin V-FITC and PI before being analysed by flow cytometry. zVAD (20 μM) was added in the culture medium 1 h prior to the addition of SeNps. (**b**) SeNps induced activation of caspase 3 in HT29 cells. SeNps-treated HT29 cells (15 μg/mL, 24 h) were fixed, permeabilised and labelled with an anti-cleaved caspase 3 antibody prior to flow cytometry analysis. zVAD was added to the culture medium, where indicated, one (1) hour before the addition of SeNps. A 20.10% ratio of cells positive for cleaved caspase 3 was observed in SeNps-treated cells compared to 1.35% in control. zVAD prevented the activation of caspase 3 by SeNps which is evident by the 0.58% ratio of cleaved caspase 3-positive cells observed in SeNps-treated cells that were also incubated with the inhibitor. Results presented are representative of at least three independent experiments. (**c**) Caspase 3 activation confirmed by fluorescence microscopy. Cells seeded on glass coverslips were treated with 15 μg/mL SeNps for 24 h, fixed, permeabilised and labelled with the same anti-cleaved caspase 3 antibody before being observed under a fluorescence microscope (magnification: 1000×). (**d**) Western blot analysis for the pro-apoptotic proteins Puma, Bax, TRAIL and the cleavage of PARP1 and caspase 3 in SeNps-treated (15 μg/mL) HT29 cells for 24 or 48 h. Note the increase in Puma, TRAIL and cleaved PARP1 as well as caspase 3 in SeNps-treated compared to control cells, for either time points, and the increase of Bax in 24 h. Western blot images presented are representative of at least two independent experiments. (**e**) Growth inhibition and M30 levels in Caco-2 cells treated with SeNps. Cell growth inhibition was analysed by an SRB assay after 24 h or 48 h of treatment. M30 levels were quantified in cell extracts. Caco-2 cells were treated with 15 μg/mL SeNps for 20–26 h. M30 levels were quantified in cell lysates with the M30 Apoptosense ELISA kit. Results in the graphs are presented as mean value ± S.D. of two independent experiments.

In order to investigate the induction of apoptotic cell death in human HT29 colon cancer cells, we employed the Annexin V-FITC/PI double-staining protocol while also using the pan-caspase inhibitor zVAD-FMK (zVAD). zVAD is a molecule that irreversibly binds to the catalytic site of caspase proteases and blocks the induction of apoptosis. As shown in Figure 2a, HT29 cells treated with 15 μg/mL SeNps for 24 h underwent apoptosis, as evident by the increase of both Annexin V-positive and PI-negative (pro-apoptotic), and Annexin V and PI-double-positive cells (necrotic) (36.4% and 24.8% in treated cells, respectively, compared to 8.4% and 13.4% in control cells). When the cells were co-treated with SeNps and zVAD, the percentages of both pro-apoptotic and necrotic cells were reduced and became comparable to the control samples comprising of cells grown in plain culture medium (10.4% and 10.8% pro-apoptotic and necrotic fractions in SeNps and zVAD-treated cells, respectively). 

We also analysed the activation of caspase 3, the most common executioner caspase during the apoptotic process. Using flow cytometry, we observed an increase of the cleaved, active form of caspase 3 in the cells treated with SeNps for 24 h, which was eliminated when zVAD was added (Figure 2b). The activation of caspase 3 in the HT29 cells treated with SeNps was further confirmed using immunofluorescence microscopy (Figure 2c), where the cells with activated caspase 3 were detected in SeNps-treated samples and, with Western blot, where a time-dependent increase of the cleaved active fragment of caspase 3 was observed in cells treated with 15 μg/mL SeNps for 21 or 24 h, accompanied by the corresponding decrease of the intact, inert caspase 3 (Figure 2d). In addition, we examined the expression of characteristic pro-apoptotic proteins with Western blot. As shown in Figure 2d we detected increased levels of the signalling molecule TRAIL, following treatment with 15 μg/mL SeNps for 24 and 48 h. Similarly, the expression of pro-apoptotic regulatory molecules Bax and Puma was also elevated, with the Puma increase being consistent in both time points, while the Bax increase was evident only after 24 h of treatment. In addition, we examined the cleavage of PARP1 as a distinctive marker of apoptotic cell death. An increased cleavage of the functional intact protein was detected in cells treated with SeNps, as evident by the identification of the characteristic fragment at 89 kDa which was more prominent at 24 h of treatment.

Furthermore, SeNps’ growth inhibitory activity was examined in the human colorectal cancer cell line Caco-2. While HT-29 cells are employed as a model of the large intestine, Caco-2 cells can be regarded as a model of the small intestine [54]. SeNps were found to inhibit Caco-2 cell proliferation rate in a concentration and time-dependent manner (Figure 2e). Moreover, the levels of the M30 epitope in Caco-2 cell extracts, that indicate caspases 3/7/9 activation and therefore apoptosis, were found to be higher in SeNps-treated cells as compared to controls (Figure 2e), reaching their peak after 23 h of treatment.

#### 3.2.2. SeNps Induce Apoptosis in CT26 Cells

**Figure 3:**
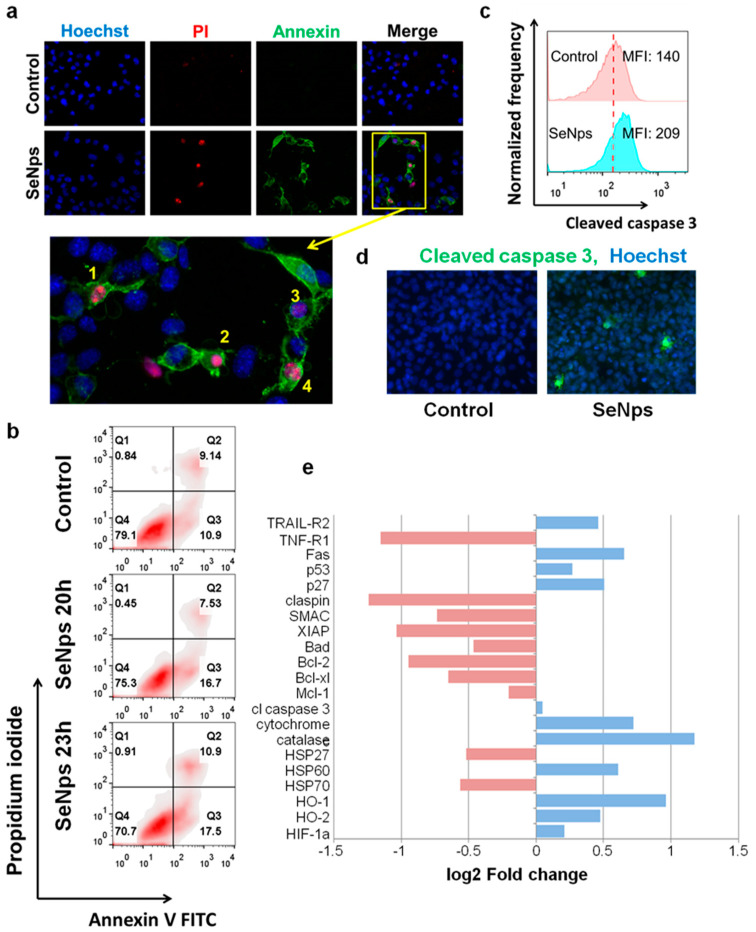
SeNps induce apoptosis in CT26 cells. (**a**) SeNps-induced morphological changes characteristic of apoptosis in CT26 cells. Cells were treated with 15 μg/mL SeNps for 23 h and stained with Hoechst (blue), Annexin V-FITC (green) and PI (red) and were examined under a fluorescence microscope. Only SeNps-treated cells appear positive for either Annexin V or PI. Magnification of the inset from the Merge fluorescence image of SeNps-treated CT26 cells illuminate morphological changes that are typical in apoptotic cells: membrane blebbing (1–4), nuclear condensation (2), nuclear fragmentation (1, 4) and membrane protrusion formation (1, 4). Figure panel is adapted from [55] (magnification: 1200×). (**b**) Flow cytometry analysis of Annexin V/PI double staining of SeNps-treated CT26 cells. Density plots of control and treated cells. The percentages of viable (Annexin V-FITC and PI-negative), early apoptotic (Annexin V-positive and PI-negative), and late apoptotic/necrotic (Annexin V/PI-double-positive) cells are indicated on the plots. Cells were treated with 15 μg/mL SeNps for 20 or 23 h and stained with Annexin V-FITC and PI before the analysis by flow cytometry. (**c**) SeNps induce activation of caspase 3 in CT26 cells. SeNps-treated CT26 cells (15 μg/mL, 24 h) were fixed, permeabilised and labelled with an anti-cleaved caspase 3 antibody prior to the flow cytometry analysis. Fluorescence intensity histograms of control cells and cells treated with SeNps and median fluorescence intensities (MFIs). The activation of caspase 3 was detected by the shift in MFI in treated cells (MFI: 209) when compared to the controls, untreated cells (MFI: 140). (**d**) Caspase 3 activation was also detected by fluorescence microscopy. Images of control and SeNps-treated (15 mg/mL, 24 h) CT26 cells labelled with Hoechst (blue, nuclei) and a secondary Alexa Fluor 488-conjugated antibody (green) against anti-cleaved caspase 3 primary antibody (magnification: 400×). (**e**) Expression of apoptosis-related proteins in CT26 cells after SeNps treatment analysed with a mouse apoptosis array (ARY031, R&D). Fold change compared to control in the expression levels of selected proteins exhibiting down (red) or up-regulation (blue) upon treatment with SeNps.

Next, we examined the induction of apoptosis in mouse CT26 colon cancer cells by SeNps with fluorescence microscopy and flow cytometry employing the Annexin V / PI double-staining protocol as described above. As shown in Figure 3a, treatment with SeNps for 23 h induced morphological changes in the cells typical of apoptosis. SeNps-treated cells exhibited membrane blebbing, nuclear condensation, nuclear fragmentation, as well as membrane protrusion formation.

Moreover, an increased percentage of pro-apoptotic and necrotic cancer cells was observed in the SeNps-treated samples stained with Annexin V-FITC/PI and analysed by flow cytometry (Figure 3b). In addition, caspase 3 was also activated in CT26 cells treated with SeNps, evident by the increased median fluorescence intensity of the treated cells when compared to controls in the flow cytometry histograms shown in Figure 3c. The presence of cancer cells with activated caspase 3 was also confirmed by fluorescence microscopy (Figure 3d). To further examine the apoptotic signalling in CT26 cells treated with SeNps, we utilised a commercially available membrane-based sandwich immunoassay which was able to detect 21 different proteins involved in various stages of apoptosis regulation. As shown in Figure 3e, two distinct apoptosis-initiating receptors, TRAIL-R2 and Fas, were increased after treatment with SeNps. In addition, both of the transcription factors p53 and p27 were elevated in the treated CT26 cells. On the other hand, the levels of checkpoint protein claspin, the apoptosis inhibitor XIAP and various pro-survival members of the Bcl-2 family, such as Bcl-2, Bcl-xl, and Mcl-1, were down-regulated. Similarly, the levels of both HSP27 and HSP70 were also decreased. Finally, cytochrome c, a cell death stimulant released from mitochondria, was elevated in cancer cells treated with SeNps.

### 3.3. Induction of Immunogenic Cell Death in CT26 and HT29 Cells by SeNps

#### 3.3.1. Release of Nuclear Protein HMGB1 from SeNps-Treated Cells

**Figure 4:**
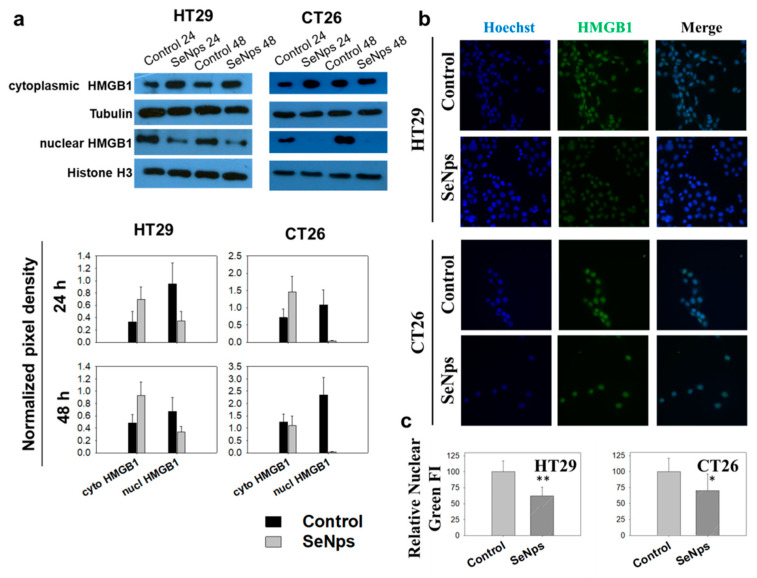
SeNps induce the release of nuclear HMGB1 from HT29 or CT26 cells. (**a**) Western blot analysis and densitometry results for the cytoplasmic or nuclear HMGB1 protein levels in SeNps-treated (15 μg/mL) HT29 or CT26 cells for 24 or 48 h. Beta-tubulin and histone H3 were used as loading controls for the cytoplasmic and nuclear fractions, respectively. Levels of nuclear HMGB1 were approximately 30 to 50% lower in HT29 cells following treatment with 15 μg/mL SeNps for 24 h, or even eliminated in the SeNps-treated CT26 cells. Cytoplasmic HMGB1 levels exhibit an approximately 100% increase in both HT29 and CT26 SeNps-treated cells. Western blot images presented are representative of at least three independent experiments. Results in graphs are presented as the mean value ± S.D. of three independent experiments. (**b**) Immunofluorescence detection of HMGB1 protein release in SeNps-treated (15 μg/mL, 24h) HT29 or CT26 cells. Fluorescence microscopy images of cells grown in IBIDI μ-slides, fixed, permeabilised and incubated with an anti-HMGB1 antibody before being labelled with Hoechst (blue, nuclei) and a secondary Alexa Fluor 488-conjugated secondary antibody (green) (magnification: 400×). (**c**) Quantification of relative nuclear fluorescence intensity (FI) indicating a statistically significant drop in both HT29 and CT26 cells pre-treated with SeNps. Results are presented as the mean value ± S.D. of two independent experiments (* *p* < 0.05, ** *p* < 0.01, Student’s *t*-test).

Our next goal was to investigate the induction of a special modality of regulated cell death able to promote an immune response called immunogenic cell death. One of the distinct main hallmarks of this type of cell death is the release of HMGB1 from the nucleus and its subsequent secretion from the dying cells [25]. As shown in Figure 4a, treatment of both HT29 and CT26 cells with 15 μg/mL of SeNps for 24 h or 48 h resulted in the release of the nuclear HMGB1, evident by the increased levels of the protein in the cytoplasm of the cancer cells and the corresponding decrease in the nucleus, an effect that for both cell lines appears to be more prominent at 24 h of treatment. The release of nuclear HMGB1 in cancer cells treated with SeNps was also confirmed using immunofluorescence microscopy. As shown in Figure 4, levels of nuclear HMGB1 were approximately 30 to 50% decreased in SeNps-treated HT29 cells when compared to control cells, while, in CT26 cells treated with 15 μg/mL SeNps for either 24 h or 48 h, nuclear HMGB1 levels were almost eliminated. It should be noted that, when either HT29 or CT26 cells were treated with SeNps (15 μg/mL, 24 h) cytoplasmic HMGB1 levels exhibited an almost two-fold increase when compared to the controls, untreated cells (Figure 4a). 

#### 3.3.2. Surface Exposure of Calreticulin and ERp57 in SeNps-Treated Cells

**Figure 5:**
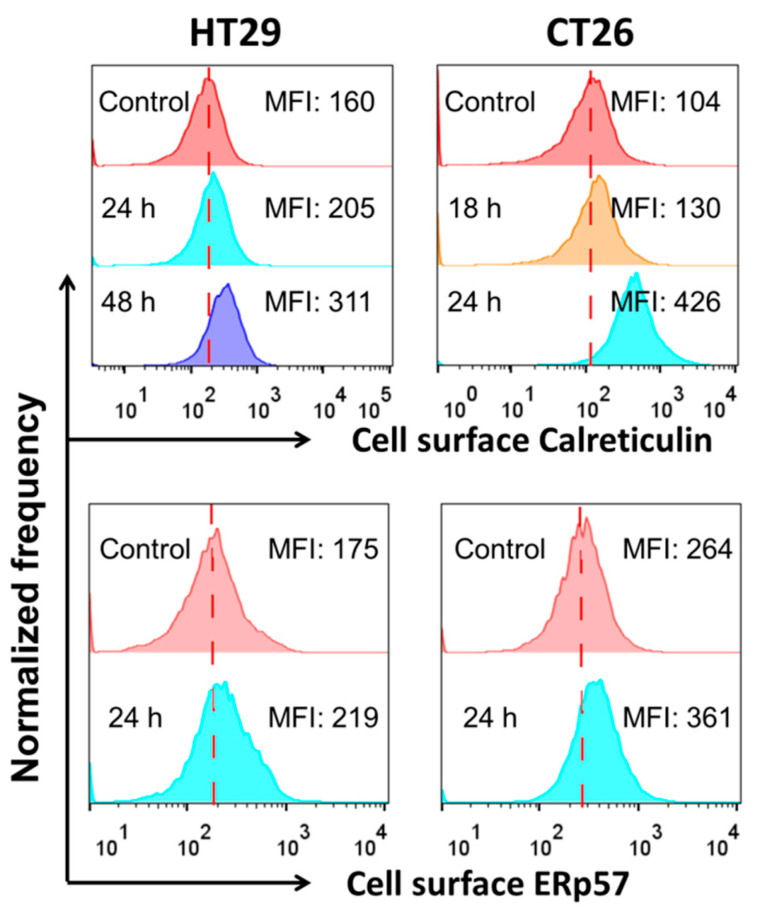
Exposure of calreticulin and ERp57 in HT29 or CT26 cells treated with SeNps. Cells were treated with SeNps (15 μg/mL) for 24 h and analysed by flow cytometry for the quantification of surface fluorescence signal, after being labelled with either an anti-calreticulin or anti-ERp57 primary antibody and a Alexa fluor-488 conjugated secondary antibody. Only live cells (7-AAD negative) were analysed; dead cells or cells with compromised membranes were excluded from the analysis by 7-AAD staining. A significant increase in the calreticulin and ERp57 exposure, evident by the elevated MFI values, was observed in both the CT26 and HT29 cells after SeNps treatment. Data were analysed with FlowJo v.10. Results are representative of at least three independent experiments. Fluorescence intensity histograms.

The second characteristic hallmark of immunogenic cell death that was examined was the exposure of the endoplasmic reticulum proteins calreticulin and ERp57 (Figure 5) on the outer layer of the cytoplasmic membrane in SeNPs-treated cells. Indeed, in both CT26 and HT29 cells pre-treated with SeNps, calreticulin and ERp57 were found to be translocated on the cytoplasmic membrane. Surface exposure of the proteins was quantified by the time-dependent increase of median fluorescence intensity (MFI) observed in treated compared to control cells. Noteworthy, the translocation of calreticulin was more prominent than that of ERp57 in both cell lines. 

#### 3.3.3. SeNps Induce the Secretion of ATP from Cells

**Figure 6:**
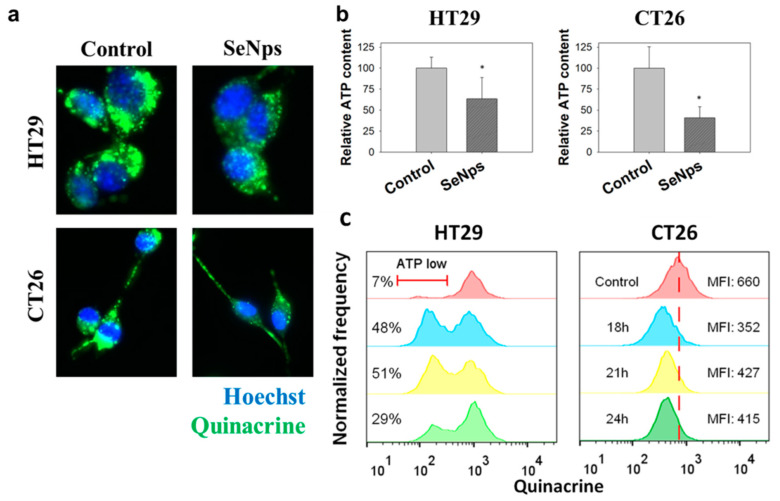
ATP secretion from SeNps-treated HT29 or CT26 cells. (**a**) Fluorescence microscopy images of ATP-containing vesicles in live HT29 or CT26 cells. Cells were grown in coverslips, treated with 15 μg/mL SeNps for 21 h and labelled with the ATP-marker quinacrine (green). A lower cellular MFI was observed in both SeNps-treated HT29 or CT26 cells when compared to the controls, untreated cells (magnification: 1200×). (**b**) Quantification of the relative cellular quinacrine fluorescence intensity, expressed as relative ATP content. A statistically significant drop in both HT29 and CT26 cells pre-treated with SeNps (* *p* < 0.05, Student’s *t*-test) was observed. (**c**) The observed lower ATP levels in SeNps-treated cells were confirmed by flow cytometry analysis. Quinacrine-labelled HT29 or CT26 cells pre-treated with 15 μg/mL SeNps for 18, 21 or 24 h were analysed in an Attune NxT flow cytometer. Fluorescence intensity histograms of cells. MFI stands for median fluorescence intensity. Data were analysed with FlowJo v.10. Results presented are representative of at least three independent experiments, (*p* < 0.05, Student’s *t*-test).

The release of ATP from dying cancer cells was examined as a third marker of immunogenic cell death. As shown in Figure 6a,b, fewer intracellular, ATP-containing vesicles were observed in the SeNps-treated HT29 or CT26 cells when compared to control cells. The relative ATP content was decreased by approximately 40 and 70% in HT29 or CT26 cells, respectively. It is interesting to note that almost all the SeNps-treated CT26 cells exhibited a reduced ATP content when compared to control, while only a portion of the HT29 cells exhibited a loss of ATP, as shown in the flow cytometry histograms in Figure 6c. 

#### 3.3.4. Secretion of Pro-Inflammatory Cytokines from SeNps-Treated Cells

**Figure 7:**
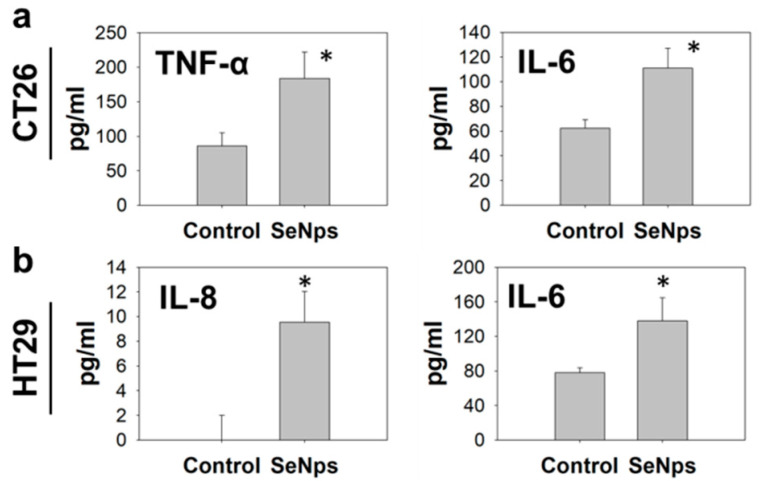
Colon cancer cells secrete pro-inflammatory cytokines upon treatment with SeNps. (**a**) Secretion of TNF-α or IL-6 from SeNps-treated CT26 cells and (**b**) IL-6 or IL-8 from SeNps-treated HT29 cells. Cells were treated with 15 μg/mL SeNps for 3 h. Quantification of cytokine levels in culture supernatants was performed by ELISA. Results are presented as the mean value ± S.D. of two independent experiments (* *p* < 0.05, Student’s *t*-test).

Furthermore, SeNps induced the secretion of IL-6 and TNF-α in CT26 cells (Figure 7a) and the secretion of IL-8 or IL-6 in HT29 cells (Figure 7b). Dying tumour cells undergoing immunogenic cell death have been reported to release such cytokines that can modulate the immune response [56,57]. 

### 3.4. Cancer CT26 Cells Destroyed by SeNps Are Preferentially Phagocytosed by RAW246.7 Macrophages

**Figure 8:**
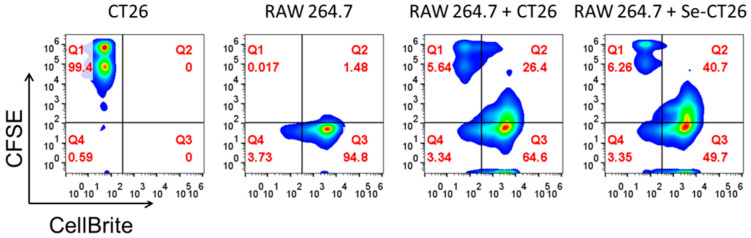
Treatment of CT26 cells with SeNps enhances the phagocytic activity of LPS-stimulated RAW264.7 macrophages against CT26-derived cellular debris. Flow cytometric quantification of phagocytosis of SeNps-treated CT26 cells by RAW264.7 macrophages. CT26 cells were labelled with CFSE prior to SeNps treatment (15 μg/mL, 48h). Treated CT26 cells and cellular debris were collected and co-cultured for 3 h with LPS-stimulated and CellBrite red-labelled RAW264.7 macrophages (RAW264.7 + Se-CT26). Moreover, RAW264.7 macrophages were co-cultured with the controls, untreated CT26 cells (RAW264.7 + CT26). Cells were collected and analysed on an Attune NxT flow cytometer. CT26 and RAW264.7 cells were also individually analysed (CT26 and RAW 264.7). An enhanced phagocytic activity was observed in macrophages co-cultured with SeNps-treated CT26 cells when compared to macrophages co-cultured with untreated control CT26 cells, evident by the elevated ratio of the double positive fraction in the analysed cell population (40.7% in RAW264.7 + Se-CT26 compared to 26.4% in RAW264.7 + CT26). Results are representative of three independent experiments.

Tumour cells develop mechanisms in order to avoid phagocytosis [58], however, cells killed by immunogenic anticancer therapies have been proposed to stimulate non-homeostatic clearance by antigen-presenting cells [59]. Herein, based on the above reported observations regarding the induction of immunogenic cell death by SeNps in colon cancer cells, we investigated whether SeNps-treated cancer cells become more prone to being phagocytosed by macrophages. For this purpose, the LPS-stimulated RAW264.7 murine macrophage model was employed, in order to assess the phagocytic activity of murine RAW264.7 macrophages against SeNps-treated CT26 cells. RAW264.7 macrophages preferentially phagocytosed SeNps-treated CT26 cells when compared to the controls, untreated cells, evident by the increased ratio of the double positive fraction (CellBrite red for macrophages and CFSE for tumour cells) observed (40.7% in RAW264.7 + Se-CT26 compared to 26.4% in RAW264.7 + CT26) (Figure 8). 

## 4. Discussion

Selenium has long been considered a promising anticancer agent due to the fact that tumour cells appear to be more susceptible to its pro-oxidant and cytotoxic activity than normal cells [12]. Selenium nanoparticles in particular stand out as an alternative to other forms for the implementation of selenium-based strategies in clinical practice on account of their unique properties, such as their reduced toxicity and improved bioavailability [7,10,12]. We have previously demonstrated that *L. casei* ATCC 393 bacteria biosynthesise selenium nanoparticles. Herein, we confirmed by TEM analysis our previous observation (by bright field microscopy) [41], that SeNps are formed intracellularly by the lactobacilli of this specific strain (Figure 1a), which is in agreement with the literature [50]. Biogenic nanoparticles, such as the SeNps studied here, have been reported to retain a capping layer consisting of biomolecules [60]. This layer contributes to the nanoparticles’ structural and chemical stability and influences their bioactivities. The layers’ composition is dictated by the organism used for the biosynthesis of the nanoparticles, while proteins, lipids, nucleic acids and carbohydrates have been proposed to be part of it [60]. SeNps, extracted from *L. casei* ATCC 3939 in particular, have been described to be enclosed in a layer consisting of proteins and polysaccharides [50]. Our results agree with the literature as we also detected small amounts of organic material in the SeNps, evident by the characteristic FTIR bands (Figure 1b). The idea that the isolated SeNps’ surface is associated with certain molecular entities derived from the bacteria is one of the main premises of using biogenic nanoparticles instead of chemically synthesised ones. The presence of such compounds generates a unique surface chemistry on the nanoparticles that prevents aggregation and leads to a more bioavailable and less toxic profile, while also affecting the cellular uptake processes. We hypothesize that the ability of SeNps to induce apoptosis and ICD in colon cancer cells, as described here, is greatly influenced by the composition of the biomolecular layer on the surface of SeNps.

We have previously described that SeNps inhibit colon cancer cell growth in vitro and in vivo, while not being toxic to primary healthy cells isolated from the colonic epithelium of mice [41]. In the present study, treatment with 15 μg/mL SeNps inhibited Caco-2 colon cancer cell growth and induced activation of caspases 3/7/9. Specifically, in CT26 and HT29 colon cancer cells, SeNps induced apoptotic mechanisms that are typically involved in the intrinsic apoptotic pathway (Figure 2 and Figure 3). It is noteworthy that the activation of the intrinsic apoptotic pathway following the internalization of SeNps and the accumulation of ROS is the most commonly described anticancer mechanism of action for SeNps [17]. We have previously described the elevated production of ROS by HT29 cancer cells treated with SeNps, where the induction of apoptosis was analysed by Annexin V-FITC/PI staining as well as the modulation of the expression of apoptosis-related proteins, such as the downregulation of survivin and cIAP-1, and the upregulation of both TRAIL death receptors, DR4 and DR5 [41]. 

Herein, we report further distinct pro-apoptotic changes in the expression of proteins of the Bcl-2 family that regulate the intrinsic pathway of apoptosis [61,62]. Both the pro-apoptotic Puma and Bax were increased in HT29 cells (Figure 2d), while in CT26 cells the antiapoptotic proteins Bcl-2, Bcl-xl and Mcl-1 were downregulated. In addition, other known apoptosis inhibitors such as XIAP, HSP27 and HSP70 were also reduced (Figure 3e). The interactions between the different Bcl-2 protein family members determine whether mitochondrial outer membrane permeabilisation occurs and if mitochondrial factors are released in the cytoplasm [61,63]. Among these factors, cytochrome c stands out as a component of the apoptosome, a protein complex that engages and activates caspase 9 [61,64], and levels of cytochrome c were found to be increased in the SeNps-treated CT26 cells in our experiments (Figure 3e). The apoptosome and caspase 9 activity is negatively affected and inhibited by XIAP [65] and HSP70 [66] which, as mentioned above, were reduced in CT26 cells treated with the SeNps. The activation of caspase 9 is a crucial step in the apoptotic process, as it enacts the caspase-cascade-activating caspases 3 and 7 [67]. Caspase 3 subsequently, through its activity as a protease, triggers cellular demise [64,65]. Both caspase 3 and cytochrome c are targets of HSP27, which negatively regulates their activity by binding them, as well as stabilizes cytoskeletal elements in order to prevent cytoskeletal disruption [66,68]. Substrates for the proteolytic activity of caspase 3 include PARP1, an enzyme responsible for DNA repair, and the cleavage of PARP1 by caspases is considered a hallmark of apoptosis, and a part of the process that culminates in the fragmentation of DNA observed during apoptosis [69]. We observed the activation of caspase 3 in both the colon cancer cell lines that were investigated after 24 h of treatment with the SeNps (Figure 2b,c and Figure 3c,d), as well as the cleavage of PARP1 to its characteristic 89 kDa fragment, which is associated with apoptosis in HT29 cells (Figure 2d). In addition, we detected the exposure of phosphatidylserine on the outer layer of the cytoplasmic membrane which is another hallmark of apoptotic cell death. It should be noted that both caspase 3 activation and the detection of apoptotic cells were reversible when the cancer cells were treated with a combination of SeNps and pan-caspase inhibitor zVAD-FMK, an observation that further supports the pro-apoptotic activity of SeNps (Figure 2a,b and Figure 3a,b).

Our observations substantiate our previously reported findings [41] and are consistent with the literature, that suggests that the main mechanism behind the direct anticancer activity of selenium nanoparticles is apoptosis [70]. Specifically, selenium nanoparticles have been shown to induce caspase-mediated apoptosis in prostate cancer cells [71], hepatocarcinoma cells [72,73], breast [73], cervical [74] and melanoma cancer cells [75]. Critically, in all of the above-mentioned papers, selenium nanoparticles were prepared by employing the chemical synthetic route. It is noteworthy that biogenic selenium nanoparticles, specifically, have been demonstrated to destroy ovarian cancer cells by triggering apoptosis [76].

In addition to triggering caspase-mediated apoptosis, we report strong indications that treatment with SeNps increases the ICD and thus, the immunogenicity in dying colon cancer cells. ROS generation, which we have previously reported, is induced by SeNps in HT29 cells [41] and ROS-mediated endoplasmic reticulum stress is required for ICD [77]. Moreover, herein, we have shown that SeNps induced the translocation of calreticulin and ERp57 to the cell surface of HT29 and CT26 cells (Figure 5). Calreticulin’s and ERp57′s translocation from the lumen of the endoplasmic reticulum to the plasma membrane is associated with ICD. Specifically, their translocation precedes apoptosis and serves as a phagocytic signal which is required for ICD [78,79]. In fact, conventional anticancer drugs, such as anthracyclines, which have been shown to induce calreticulin and ERp57 translocation to the cell surface, induce ICD in cancer cells [80,81]. 

Among the main hallmarks of ICD, other than the surface exposure of calreticulin and ERp57, is the release of HMGB1 and ATP [25] from cells. Nuclear HMGB1 passes through the permeabilised nuclear lamina, first into the cytoplasm and is finally released extracellularly in cells undergoing ICD [25,82], while ATP is released to the extracellular space through an autophagy and caspase-dependent manner [83]. In both CT26 and HT29 cells that were treated with SeNps, a significant drop in HMGB1 nuclear content was observed, accompanied by an elevation of HMGB1 levels in the cytosol, indicating the release of HMGB1 from the nucleus to the cytosol (Figure 4). Moreover, lower ATP levels were detected in both cell lines following treatment with the SeNps (Figure 6). These findings indicate that SeNps trigger the exposure and release of the main ICD-associated DAMPs, that, along with the activation of additional ICD-related mechanisms, are able to dictate the ability of dying cancer cells to trigger an adaptive immune response [22]. 

Another observation that further supports the ICD-inducing capability of SeNps was the secretion of IL-8 and IL-6 from the SeNps-treated HT29 cells, and IL-6 and TNF-α from the SeNps-treated CT26 cells (Figure 7). Cancer cells undergoing ICD release pro-inflammatory cytokines that can regulate host immune responses [57]. Pro-inflammatory cytokines such as Il-6 and TNF-α, when released following the induction of ICD, can induce MHC class I expression on antigen-presenting cells and promote T-cell differentiation and NK cell activation [57,84]. Interestingly, the cytokines IL-6, IL-8 and TNF-α that were detected in the supernatants of the SeNps-treated cells, have been previously reported to be released by cancer cells undergoing ICD and to be involved in the signalling of dendritic and natural killer cells [22,56,85].

The secretion of pro-inflammatory cytokines, the extracellular release of HMGB1 and ATP, and the surface exposure of calreticulin and ERp57 consist of the main biomarkers for the induction of ICD and operate as “find me” signals for the induction of the clearing of dying cancer cells by phagocytosis, eliciting an immune response [25,57,86]. In our experiments, all the above ICD hallmark processes were identified in SeNps-treated colon cancer cells. In order to further explore the potential immunostimulatory activity of SeNps suggested by the current findings, we proceeded to examine whether macrophages demonstrate enhanced phagocytic activity against SeNps-treated cancer cells. As shown in Figure 8, an approximately 14% elevation was detected in the phagocytic capability of RAW264.7 murine macrophages against SeNps-treated CT26 cells when compared to control cells. This observation is an extra step towards the verification of the translational potential of *L. casei* ATCC 393-biosynthesised SeNps, for ICD induction in cancer cells. 

## 5. Conclusions

The present study highlights the pro-apoptotic activity of biogenic SeNps, synthesised by *L. casei* ATCC 393, and their potential to promote immunogenicity in dying colon cancer cells. We had previously reported that this probiotic bacterial strain synthesises selenium nanoparticles, under specific conditions, that exert cancer-specific antiproliferative activity against colon cancer cells, induce apoptosis-related morphological changes, modulate apoptosis-related protein levels, and inhibit tumour growth upon oral administration, in a syngeneic CT26 colon tumour model in BALB/c mice [41]. The aim of the current study was to further investigate the potential pro-apoptotic effects exerted by SeNps in colon cancer cells and to examine whether SeNps are able to induce ICD. Our results add to the growing evidence that SeNps induce apoptosis in cancer cells. More specifically, we describe that SeNps induce caspase-dependent apoptosis in HT29 or CT26 colon cancer cells. This conclusion is supported by (i) the increased number of pro-apoptotic SeNps-treated cells accompanied by caspase 3 activation in both cell lines and, the lack of both pro-apoptotic cells and the presence of cleaved caspase 3 when the SeNps-treated cells were simultaneously incubated with a pan-caspase inhibitor and, (ii) the modulation of the expression levels of various apoptosis-related proteins such as the elevated cleavage levels of PARP1, the upregulation of TRAIL, TRAIL-R2, Fas, Bax, Puma, p53 and p27 and the downregulation of various protein members of the pro-survival Bcl-2 family. Our findings also indicate that SeNps could affect the immunogenicity of dying colon cancer cells. Crucially, we detected some of the main biomarkers of ICD, namely, the translocation of calreticulin/ERp57, the release of HMGB1 and ATP, and the secretion of pro-inflammatory cytokines from the SeNps-treated dying cancer cells. Taken together, these observations indicate that the direct anticancer effects induced by SeNps could be enhanced by the development of an efficient immune response. Thus, treatment with SeNps, might be an efficient strategy to destroy tumour cells in the development of new therapeutic approaches against cancer, including tumour-cell-based vaccines. 

Tumour-cell-based vaccines comprise syngeneic cancer cells, or cells derived from allogeneic cell lines that are being treated in order to be inactivated and for their immunogenicity to be enhanced, before being administered to the patient [87]. Such vaccines are among the earliest vaccines investigated against cancer [87] and have showed very promising effects in preclinical mouse tumour models but, unfortunately, they did not elicit the same extent of therapeutic efficacy in the various clinical trials that followed [88]. Thus, new ICD-inducing agents should be explored. Nowadays, more DAMPs have been recognised in various cell-treatment systems and novel compounds have been used for the induction of ICD, such as various types of nanoparticles [89,90]. Moreover, microorganisms such as yeast and bacteria have been used along tumour vaccines to stimulate an immune response [88]. Our team has previously described that the bacterial strain used here for SeNps synthesis, *L. casei ATCC* 393, induced potent Th1 immune responses and cytotoxic T-cell infiltration in the tumour tissue of CT26 tumour-bearing mice, resulting in tumour growth inhibition [91]. Since SeNps are being synthesised intracellularly, the combination of SeNps and *L. casei* or even SeNps alone might possess a dynamic potential in being utilised for the development of a cancer-cell based vaccine. Furthermore, having observed the profound effects of orally administered SeNps in the CT26-tumour model in mice [41], we believe that SeNps could be used in oral formulations alongside traditional anticancer approaches, such as chemotherapy, radiation therapy and surgery, in the development of novel combined-modality treatment approaches. 

However, there are certain limitations in the interpretations of our results, the main one being that our experiments regarding ICD presented here were only conducted in in vitro models. Future research will be focused on the in vivo investigation of the potential immunostimulatory effects of SeNps, or SeNps and *L. casei* treatment, against colon cancer in the CT26 mouse tumour model. We believe that biogenic SeNps derived from *L. casei* ATCC 393 hold great promise as a novel anti-tumour agent that induces ICD.

## Data Availability

Supporting data are available from the authors upon request.

## Supplementary Materials

The following are available online at www.mdpi.com/article/10.3390/cancers13215335/s1, Figure S1: Gating strategy for flow cytometry analysis of ATP intracellular levels and surface exposure of calreticulin or ERp57 in HT29 and CT26 cells, Figure S2: Western blot images for CT26 cells and images of dot plots of protein levels analysed with a mouse apoptosis array, Figure S3: Western blot images for HT29 cells, Figure S4: μ-FTIR spectra of *L. casei* ATCC 393 bacteria grown in their standard medium or in the presence of 20 μg/mL NaHSeO_3._
