# Supplementary material for: Anticancer Activity of Biogenic Selenium Nanoparticles: Apoptotic and Immunogenic Cell Death Markers in Colon Cancer Cells
Source: Cancers (Basel). 2021 Oct 24;13(21):5335. doi: 10.3390/cancers13215335 (PMC8582357; doi:10.3390/cancers13215335)
Supplement: Supplementary file 1 [file cancers-13-05335-s001.zip › cancers-1388368-supplementary.pdf]

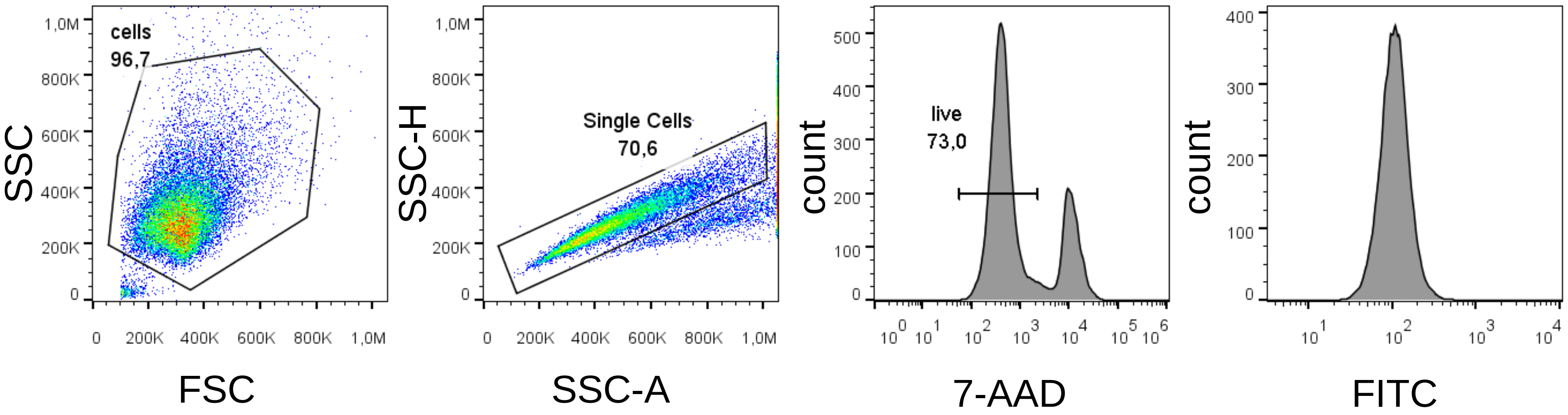

Figure S1. Gating strategy for flow cytometry analysis of ATP intracellular levels and surface exposure of calreticulin or ERp57 in HT29 and CT26 cells. Cells were incubated with 4 µM quinacrine dihydrochloride (for the quantification of ATP levels) or labeled with either an anti-calreticulin or anti-ERp57 primary antibody and a Alexa fluor 488 conjugated secondary antibody. Dead cells or cells with compromised membrane were excluded from the analysis by 7-AAD staining. Only live cells (7-AAD negative) were analyzed.

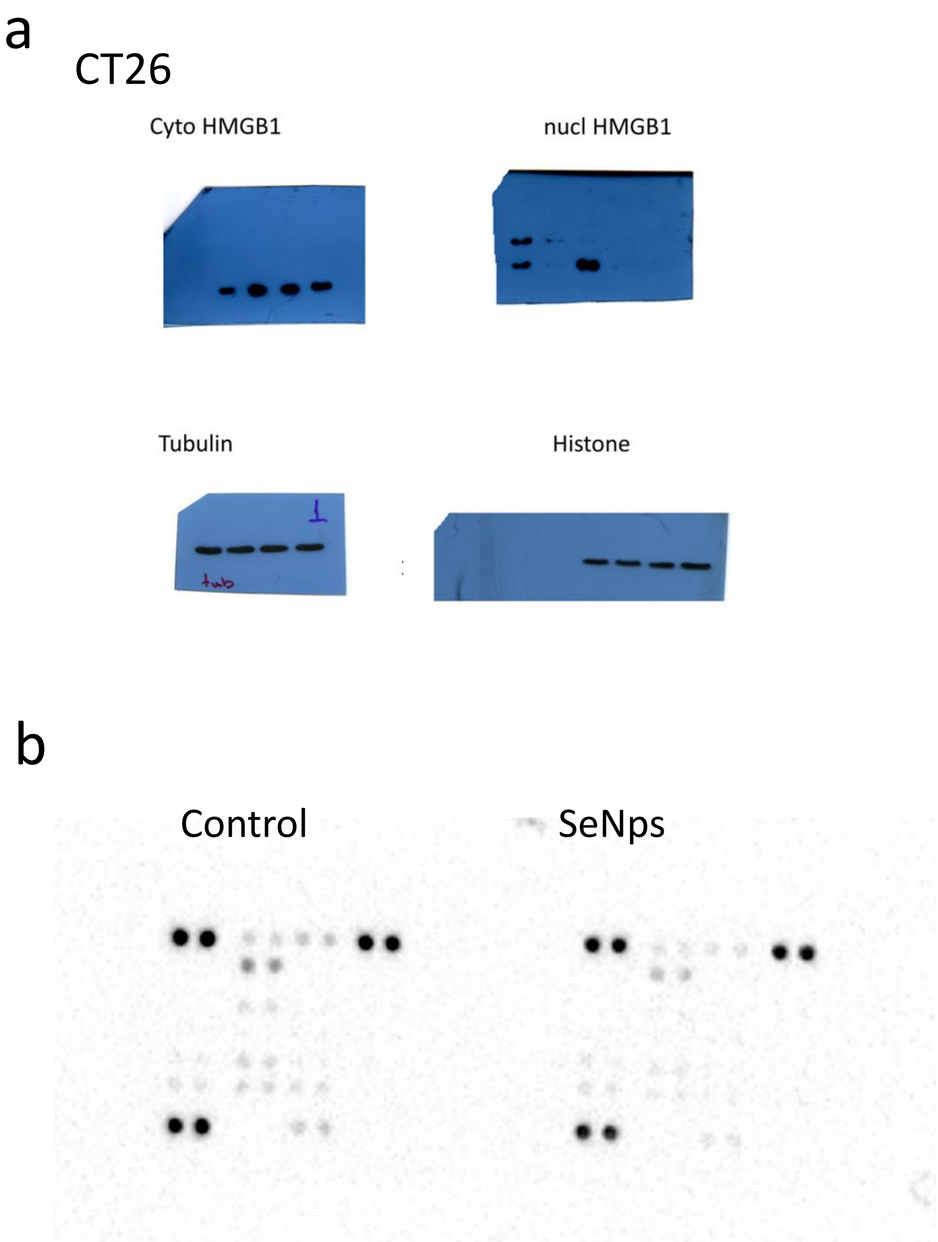

Figure S2. (a) Western blot images for CT26 cells. (b) Images of dot plots of protein levels analyzed with a mouse apoptosis array (ARY031, R&D Systems).

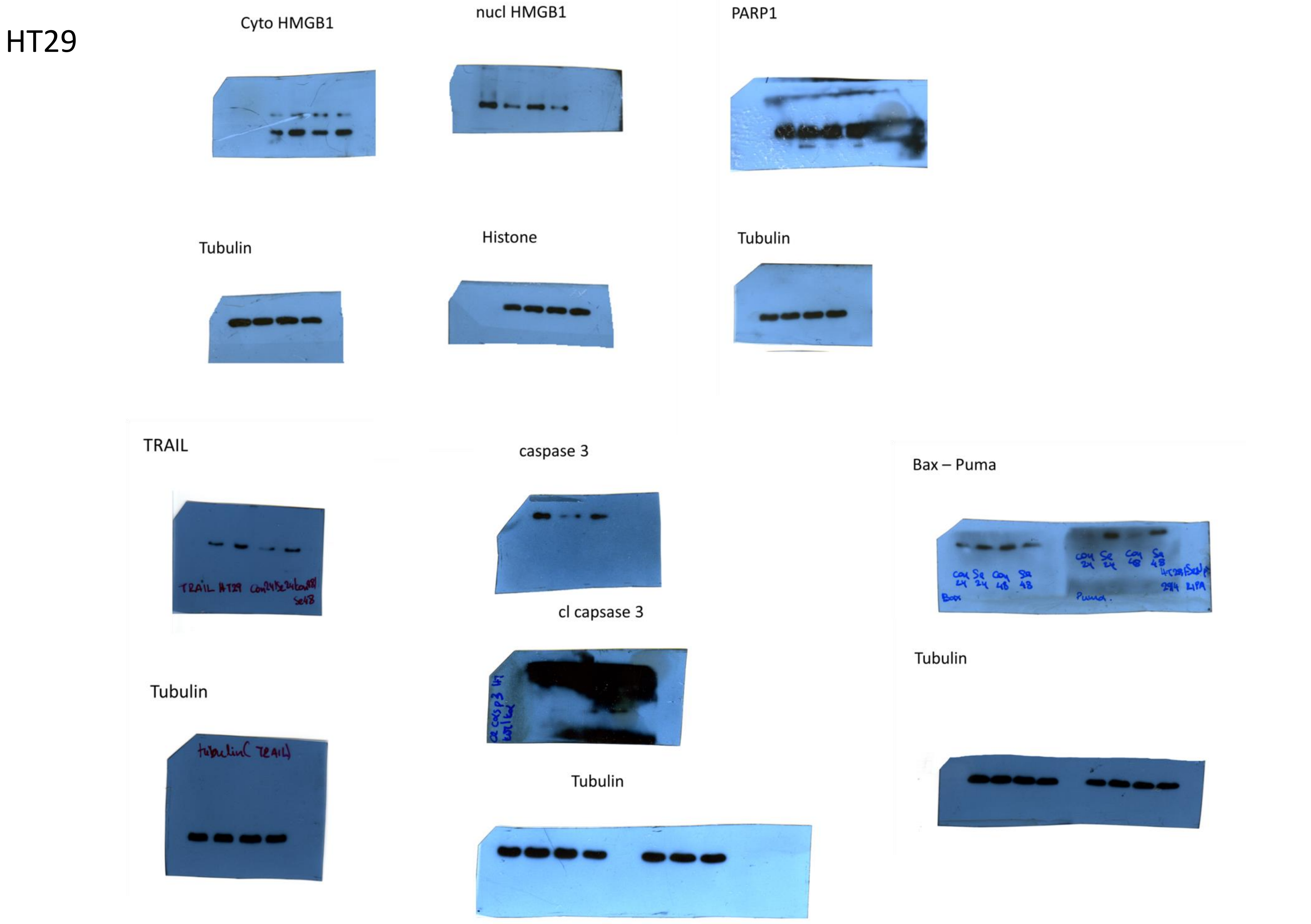

Figure S3. Western blot images for HT29 cells.

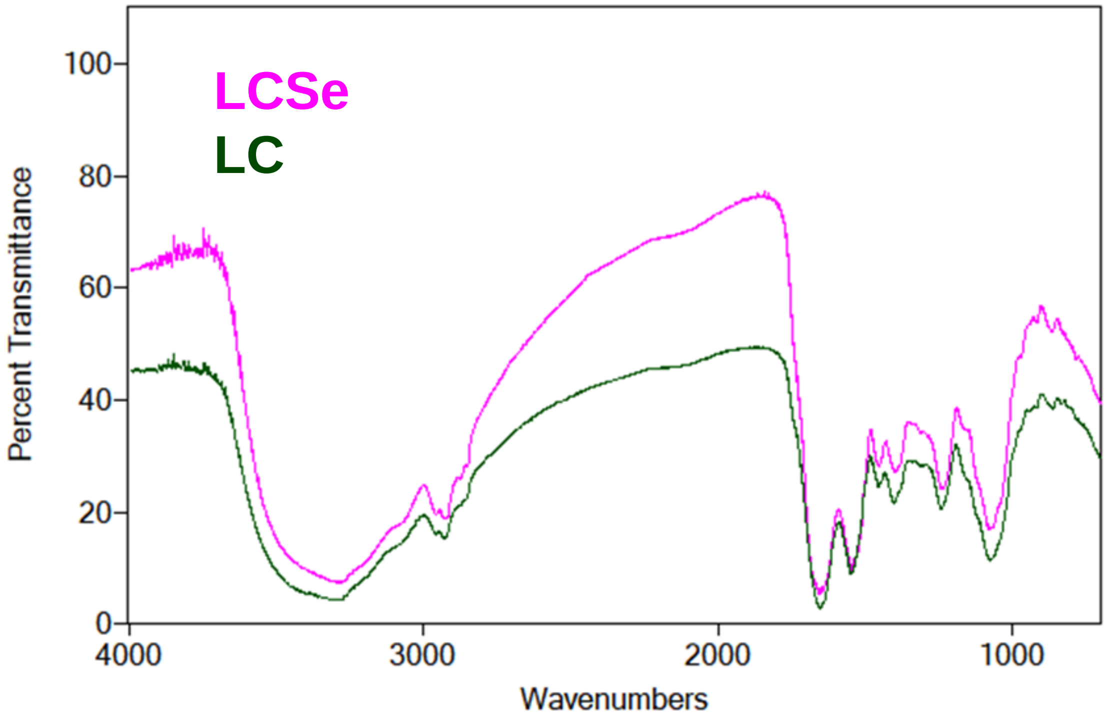

Figure S4. µ-FTIR spectra of *L. casei* ATCC 393 bacteria grown in their standard medium (LC, green line) or in the presence of 20 µg/ml $NaHSeO_3$ (LCSe, purple line).
